# Preventing Peer Violence Against Children: Methods and Baseline Data of a Cluster Randomized Controlled Trial in Pakistan

**DOI:** 10.9745/GHSP-D-16-00215

**Published:** 2017-03-24

**Authors:** Judith McFarlane, Rozina Karmaliani, Hussain Maqbool Ahmed Khuwaja, Saleema Gulzar, Rozina Somani, Tazeen Saeed Ali, Yasmeen H Somani, Shireen Shehzad Bhamani, Ryan D Krone, Rene M Paulson, Atta Muhammad, Rachel Jewkes

**Affiliations:** a Texas Woman's University College of Nursing, Houston, Texas, USA.; b The Aga Khan University School of Nursing & Midwifery, Karachi, Pakistan.; c Elite Research, LLC, Irving, Texas, USA.; d Right To Play, Hyderabad, Pakistan.; e South African Medical Research Council, Pretoria, South Africa.

## Abstract

**Background:**

Violence against and among children is a global public health problem that annually affects 50% of youth worldwide with major impacts on child development, education, and health including increased probability of major causes of morbidity and mortality in adulthood. It is also associated with the experience of and perpetration of later violence against women. The aim of this article is to describe the intervention, study design, methods, and baseline findings of a cluster randomized controlled trial underway in Pakistan to evaluate a school-based play intervention aiming to reduce peer violence and enhance mental health.

**Methods:**

A cluster randomized controlled design is being conducted with boys and girls in grade 6 in 40 schools in Hyderabad, Pakistan, over a period of 2 years. The Multidimensional Peer-Victimization and Peer Perpetration Scales and the Children's Depression Inventory 2 (CDI 2) are being used to measure the primary outcomes while investigator-derived scales are being used to assess domestic violence within the family. Specifics of the intervention, field logistics, ethical, and fidelity management issues employed to test the program's impact on school age youth in a volatile and politically unstable country form this report.

**Baseline Results:**

A total of 1,752 school-age youth were enrolled and interviewed at baseline. Over the preceding 4 weeks, 94% of the boys and 85% of the girls reported 1 or more occurrences of victimization, and 85% of the boys and 66% of the girls reported 1 or more acts of perpetration. Boys reported more depression compared with girls, as well as higher negative mood and self-esteem scores and more interpersonal and emotional problems.

**Interpretation:**

Globally, prevalence of youth violence perpetration and victimization is high and associated with poor physical and emotional health. Applying a randomized controlled design to evaluate a peer violence prevention program built on a firm infrastructure and that is ready for scale-up and sustainability will make an important contribution to identifying evidence-informed interventions that can reduce youth victimization and perpetration.

## Abstract (precis)

Peer violence was remarkably high at baseline. Among urban public school students, 94% of 6th-grade boys and 85% of girls reported being victimized by peers in the last 4 weeks. And 85% of boys and 66% of girls reported perpetrating such violence. Boys scored worse on a number of mental health measures. A cluster RCT is underway to evaluate a well-established school-based intervention using sports and games to reduce peer violence.

Violence against children is a global public health problem, affecting 50% of youth worldwide each year.^1^ It takes many different forms, of which violence among children (also known as peer violence or bullying) and violence by caregivers against children (including child sexual and physical abuse) are the most commonly described. Most research comes from high-income countries, but in recent years there has been an increasing focus on documenting and developing responses to the problem in a more global context, led in particular by Together For Girls, a public-private partnership of several U.S. Government agencies and 5 United Nations (UN) partners headed by the United Nations Children's Fund (UNICEF).^2^

Violence against children violates children's human rights and impacts their education, quality of life, and mental health and dramatically increases the probability of major causes of morbidity and mortality in adulthood.^3^^–^^5^ Furthermore, violence experienced by children or perpetrated by children is associated with experience of and perpetration of later violence against women, as well as other adult violence, and impacts children's ability to reach their full social and economic potential in adulthood.^5^ The 2030 Agenda for Sustainable Development^6^ highlights the importance of securing youth safety for global development by requiring the prevention of all forms of violence against children, which is further endorsed by the UN report on violence prevention.^7^

Evidence demonstrates that drivers of violence against children are found at different levels of the socioecological model within the child, family, community/school, and society.^8^ In the area of prevention of youth peer violence, which is the focus of this article, interventions have largely been school-based (with or without involvement of families) or skills/cognitive behavior modification-based, and some have included work on focal groups, such as bystander behavior.^9^ The theoretical basis for school-wide interventions starts with the view that a school itself is an ecosystem. The logic for this ecological approach is particularly strong for most peer violence occurs when traveling to or from school or in the school. Within the school, behaviors and attitudes are influenced by psychological and social factors within or related to the child; attitudes, behaviors, and lessons within the peer environment and conveyed through the teachers and principal; and the broader school policy and social context that includes formal policies, management style, and attitudes toward the use of violence. A recent systematic review identified 17 published randomized controlled trials (RCTs) of interventions to prevent peer violence, all of which were conducted in Australia, Europe, or the United States.^9^ In general, whole-school interventions have been shown to be more successful than focused interventions.

This article describes the methods of an evaluation that is being undertaken as part of the "What Works to Prevent Violence?" global program,^10^ funded by the UK Department for International Development (DFID). The initiative seeks to learn what works to prevent violence against women and girls in low- and medium-resourced countries. The program held a competitive grant process that sought to identify ongoing violence prevention initiatives located in Africa, Asia, and the Middle East that were already being delivered at scale or had good potential for scale-up and sustainability but had never been rigorously evaluated. One such program selected, which forms the focus of this research, is "The Positive Child and Youth Development" program of the Right To Play^11^ Pakistan office.

This article describes the methods of an evaluation underway to assess the effectiveness of a peer violence prevention initiative.

**Figure:**
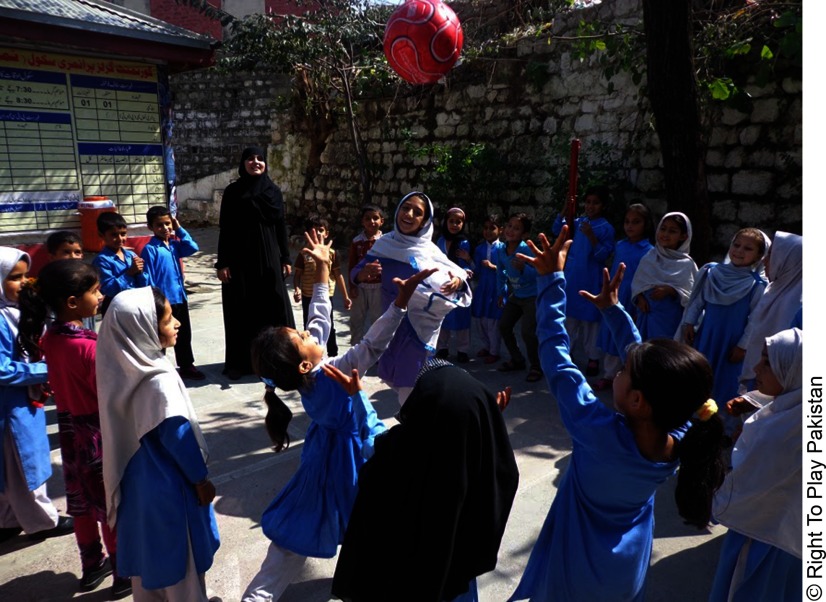
Students in Pakistan engage in the Right To Play curriculum through games and activities twice weekly.

Right To Play is an international NGO that has worked with more than a million children in 20 countries using the transformative power of play to build essential life skills, enhance school retention, and prevent violence among children. Specific to violence prevention, the Right To Play objectives posit a change in social norms that contribute to violence against women and girls, especially attitudes that support gender inequities and subordination of girls and women. Additionally, the program aims to empower girls and boys to prevent interpersonal violence and simultaneously build the capacity of schools, teachers, education departments, and communities to reduce violence against women and girls. Right To Play is operating in many countries but has not previously been evaluated with an RCT.

Right To Play's Positive Child and Youth Development program in Pakistan includes games and activities from the manual *Red Ball Child Play* that focus on 4 areas of youth development, including physical, cognitive, social, and emotional components.^11^ These structured activities, designed to help children and adolescents improve their confidence, resilience, and critical thinking, were developed by a team of experts including educationists, athletes, teacher-trainers, and psychologists. All the games were designed to meet specific learning outcomes for particular age groups of children, and then these games are compiled in the form of manuals. The games can be modified at the field level. For example, a game can be modified according to setting and environment to include a child with a disability.

These *Red Ball Child Play* and youth development activities are integrated into the school schedule through a 35–40-minute time period twice weekly. A monthly schedule for each game session is discussed within Right To Play's implementation team, the school head, and teachers. Depending on the need of the school or curriculum outcome, a game is selected and children are engaged in it by the Right To Play coach and/or teacher during these periods. For community-based programs, a mutually agreed upon timeslot is allocated to conduct these games at community settings. The Positive Child and Youth Development program has been delivered to more than 200,000 children in Pakistan.

This article outlines the methodology, issues, concerns, and essential processes of a cluster RCT to test impact on violence prevention outcomes of the Positive Youth and Child Development program that is being delivered to low-resourced children in public, urban school settings in Hyderabad, Sindh province, Pakistan.

## METHODS

### Study Purpose and Objectives

The primary objective of this RCT is to determine whether exposure to sport and play as practiced by Right To Play's intervention is effective in reducing experience and perpetration of violence among children and enhancing mental health among boys and girls in grade 6. The secondary objectives are to determine whether the exposure to the intervention is effective in improving school performance and attendance, reducing exposure to violence at home and corporal punishment at school, and changing gender attitudes, including attitudes toward violence, among boys and girls in grade 6.

### Study Design

The study used a cluster RCT design with 2 arms. The intervention arm is receiving the full Positive Child and Youth Development program of Right To Play delivered over 2 years, beginning in January 2016 and ending in December 2017, while the control arm receives regular schooling.

### Population and Setting

The study is set among 6th graders in 40 public schools in Hyderabad, Sindh province, Pakistan. We chose Sindh province for ease of access for the research team and because Right To Play has been active there for some years. Hyderabad is an accessible city (3 hours' drive from Karachi, Pakistan), large enough to give the requisite number of clusters for the study, and one in which Right To Play had not previously worked.

We selected the 6th grade for several reasons. Peak school-age perpetration and victimization occurs between the ages of 13 to 15 years.^12^ Therefore, to prevent violence, an intervention is required during the pre-teenage years. In Pakistan, middle school includes grades 6–8, and so children recruited in 6th grade theoretically would be followed fairly easily for the 2 years of the trial, before leaving school completely or, if fortunate, entering a high school. Thus, we focused on initiating the intervention with 11–12-year-olds in the 6th grade and continuing through the 8th grade for maximum prevention impact. Public schools were selected to increase potential scale-up and integration of Right To Play's intervention, if proven effective, into the public, government-sponsored system of education in Pakistan.

Peak school-age perpetration and victimization occurs between the ages of 13 to 15 years, so interventions to prevent peer violence should start during the pre-teens.

Access to the schools for the research was given at 2 levels of educational administration: each individual school head Master or Mistress and the district-level administrator. We needed 40 fairly homogenous schools from which to randomize that met the criteria of having a play area where the program could be implemented and with school directors who were willing to participate and commit time during the school day, twice a week, to the intervention sessions. We identified 50 gender-specific schools that met all criteria and were willing to participate. We visited each school to assess willingness to participate, examine school facilities to assess a safe play area, and collect information on school attendance to ensure an adequate cluster sample size of youth from each school.

Inclusion criteria for schools were thus to be single-gender public secondary schools with an outside playground or indoor space in which games could be played, and to have 35 or more students in the grade-6 class who would give consent to participate. To reduce contamination between arms, if there were more than 40 eligible schools we included only schools that were more than 1 kilometer away from the nearest other included school of that gender.

The inclusion criteria for children were that they be students in grade 6 in selected schools, obtain consent for the study from their parents, and agree to participate themselves. The youth needed to read the national language Urdu or provincial language Sindhi competently, so they could self-complete the questionnaire. Where schools had fewer than 50 children in a grade-6 class, we approached all grade-6 classes to be in the study and accepted children who gave consent. If there were more than 50 grade-6 children, we randomly selected a grade-6 section (or 2 grade-6 sections) at the school to get a number as close as possible to 35 to invite for the study. All the children in grade 6 receive the intervention, but not all are part of the research.

### Power Analysis and Sample Size

The study has 3 primary outcomes: the mean score on a scale measuring victimization of violence among children over the past 4 weeks; the mean score on a scale measuring perpetration of violence among children over the past 4 weeks; and the mean score on a scale measuring self-reported child depression over the past 2 weeks. All outcomes will be measured at 24 months post-baseline. We recognize that in the past primary outcomes have tended to be more narrowly defined as single measures, but there is a well-established precedent when evaluating interventions that seek to achieve multiple results that are equally important to select a small number of primary outcomes.^13^^–^^16^

The study assesses outcomes at 3 levels: peer victimization, peer perpetration, and child depression.

A cluster RCT was required for intervention testing at the school level. A literature review revealed a small expected effect size of 0.2 difference between the mean scores of youth peer victimization and perpetration scores between intervention and control arm schools, following effective violence prevention interventions.^17^ *A priori* power analysis was conducted to determine the minimum sample size and cluster sizes required to find significance with power set at 0.80, an alpha level of 0.05, and a small effect size of 0.20 (f).^18^ Based on the analysis, it was determined that a minimum of 25 students per school and 20 schools per treatment and control groups were required to ensure adequate power for a hierarchical linear model.^19^

This sample and cluster size is adequate for binary and continuous dependent measures. Schools were randomly selected and then randomly assigned to either the treatment or control group. Since schools are segregated by gender, we needed 20 schools in the intervention group (10 boys' schools and 10 girls' schools) and 20 schools in the control group (10 boys' schools and 10 girls' schools). Student data were needed on a minimum of 1,000 students, 25 youths in each school, collected across 40 schools and measured yearly for 3 consecutive years to compose baseline, 12-month outcomes, and 24-month outcomes.

We learned about 20% to 30% of 12-year-old youth drop out from school, usually to marry, migrate, or join the labor force, and would therefore be unavailable to complete a 2-year study. Allowing for up to 40% attrition, we added a minimum cluster size of 35 youth per school for a minimum sample size of 1,400.

### The Positive Child and Youth Development Program

The intervention is being delivered to all children in the selected schools, even though not all students are included in the research. This is in keeping with a whole-school approach and best practices with regard to school interventions.^9^ Although the approach of using the transformative power of play has not been one that has been previously evaluated, the intervention could be classified as belonging to the family of interventions that seek to more generally build social and emotional capabilities in children (such as the Positive Action Program^20^), rather than focusing on addressing bullying per se and establishing bullying policies (an example of the latter being the KiVa programme^21^). Interventions within both of these categories, especially Positive Action and KiVa, have been shown to be effective, but they have not been rigorously evaluated in a setting akin to Pakistan.

Male and female coaches deliver the intervention. Criteria for coach selection include completion of an intermediate education, previous experience in working with children, a passion and willingness to participate in Right To Play's training, a positive attitude toward child protection, and living in relatively close proximity to the school where the coaches will work. The latter is advised due to scarcity and unpredictability of available public transportation. Coaches and project staff meet twice a month for discussion of issues and challenges and further training if appropriate. Coaches are also trained to identify and mentor junior youth leaders in the schools.

The intervention for the youth follows the age-specific *Red Ball Child Play* manual. For the age group of the study participants, there are 103 learning games (activities) in the manual, which will be imparted over 2-years' time through 130 learning sessions. The sessions are organized into 5 thematic groups (known as balls) (Table 1). Red Mind Ball games are designed to enhance concentration skills among children and develop organizational skills, which help children to learn strategic thinking. Games in the Black Body Ball focus on physical development, while games with the Yellow Spirit Ball are designed to develop positive emotions, self-confidence, and hope, and to overcome negative emotions. Blue Peace Ball games are designed to promote positive emotions and control negative emotions to build healthier personalities of children. Finally, Green Health Ball games are designed to sensitize and educate children about well-being by providing knowledge and strategies to ensure good hygiene. The play-based learning activities are offered twice a week during 40-minute sessions by the coaches, who follow a curriculum of games and discussions. Each 40-minute session includes time for the students to participant in the play-based activity and then discuss to reflect, connect, and apply the content. For examples of games and discussion formats from the Blue Peace Ball and Yellow Spirit Ball, see the supplementary material.

**TABLE 1.**
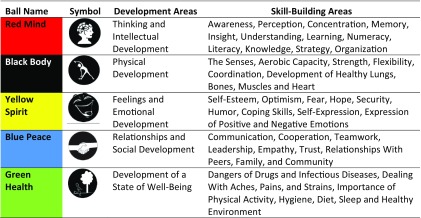
Development and Skill-Building Areas Addressed by the *Red Ball Child Play* Activities by “Ball” Thematic Groups

The games are designed for play with fairly basic equipment. Further, most can be played in an indoor room, which is a requirement in some schools for girls and essential at some times of year due to heat. However, all the schools included in the study have some form of outside area in which the games can be played. This ranges in practice from a large interior courtyard to a relatively small walled space on the roof of a school. Initially the games are led by the trained coaches and later by junior leaders selected from among the children. Junior leaders are given leadership training, and they participate as assistants to the coaches, for example, by leading warm-up exercises. Sixty junior leaders (30 boys and 30 girls) were trained in accordance with Right To Play's Junior Leader Facilitation Toolkit.

Right To Play's intervention goes well beyond the *Red Ball Child Play* manual in its efforts to provide change through holistic engagement. There are also sports tournaments and thematic Play Days (for example, focused on the theme of "Stop Violence") held several times a year (each attended by about 400 people), and parents are invited to engage in these events. They serve to increase the visibility, in particular, of girls' engagement in sport. There is also selection of youth ambassadors (10 girls and 10 boys) for training on community sensitization and mobilization to prevent violence against women and girls. Youth ambassadors are the volunteer youth from the local communities who are passionate about bringing positive change in their communities and becoming active change-makers. They are provided with mentorship and leadership training by Right To Play in order to strengthen them in skills of leadership, gender equality, communication, action planning, team work, and the role of sport and play for youth development. After attending the training, these youth ambassadors go back to their communities, identify pressing challenges, and implement small-scale projects to tackle the ground-level issues, such as making safe areas for play.

The school-based intervention also engages parents and the community at large through tournaments, events, and other activities.

**Figure:**
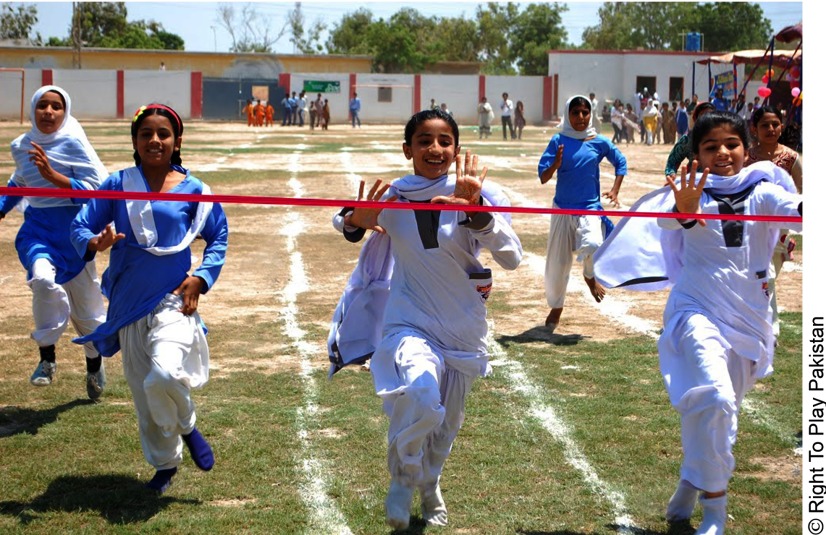
The Right To Play intervention in Pakistan encouraged girls' engagement in sports by holding tournaments and community events.

In addition, Right To Play has a network of community groups and holds quarterly awareness sessions with them, including parents, on child rights, gender equality, and positive discipline. Further, there is training of teachers on Right To Play's foundational resources, positive disciplining, and gender and child protection in order to create a safer environment in and around schools. For example, midway through the first year of the intervention, 2 summer camps were held (1 camp for boys and 1 camp for girls) with the theme "Inclusion, Friendship, Equality, and Peace." Right To Play worked closely with the District Education Office who participated in the event alongside teachers, parents, and local community-based organizations. More than 200 children aged 11–14 years (50% girls) attended the camps.

The activities of Right To Play to which research participants will be exposed will continue on an ongoing basis between the start of grades 6 until the endline assessment. At that point, the intervention will then be delivered to control arm schools.

### Logistics of Randomization

A public randomization of intervention and control schools was conducted to build trust and increase transparency of the research project. We invited all officials in the school district to the draw and gave them time to introduce themselves, their role, and name their school and location. We then followed the presentations with the public random draw. All schools signed an agreement of participation. Understanding all schools might desire the intervention, we offered 6 months of the intervention to all control schools following the final outcome measures. Further, we mitigated the disappointment of being a control arm school by offering control schools a water tank as an incentive. We chose this after consulting with school partners and parents and learning that all public schools in the area had a pressing need for potable water. Many schools did not have a water tank, resulting in dehydration among some youth who could not bring their own drinking water to school and the frequent need to dismiss school early due to lack of potable water.

### Instruments

Instruments were selected following data analysis for the formative phase and in alignment with the primary outcomes of reducing youth perpetration and victimization and the secondary outcome of improving child mental health. Toward this end, we chose Multidimensional Peer-Victimization and Peer Perpetration Scales^22^ and the Children's Depression Inventory 2 (CDI 2).^23^ For food security, gender attitudes, and family life, investigator-initiated questionnaires were developed. Table 2 presents a description of all instruments used along with coefficient alpha. All instruments were forward-translated from English to Urdu and Sindhi. People who had not seen the English questionnaire and had not participated in the forward translation independently back-translated the instruments. Discrepancies were discussed between the translators and resolved until language agreement was reached.

**TABLE 2.** Instruments to Measure Primary and Secondary Outcomes of the School-Based Positive Youth and Child Development Program, Hyderabad, Pakistan

Scale/Assessment	Characteristics	Alpha for Present Study
**School Victimization and Perpetration**		
Multidimensional Peer-Victimization Scale^22^	16-item measure with 4 subscales assessing physical and verbal victimization, social manipulation, and property attacks. Point values are assigned to responses: never=0; once=1; a few times=2; many times=3. Scale scores summed to a possible range of 0 to 48.	Peer victimization overall=0.873 Physical=0.673 Verbal=0.642 Social manipulate=0.696 Property attacks=0.658
Peer Perpetration Scale^22^	16-item measure with 4 subscales assessing physical and verbal perpetration, social manipulation, and property attacks. Point values are assigned to responses: never=0; once=1; a few times=2; many times=3. Scale scores summed to a possible range of 0 to 48.	Peer perpetration overall=0.890 Physical=0.733 Verbal=0.696 Social manipulate=0.723 Property attacks=0.716
**Location and Impact of Victimization**		
Peer Victimization Location and Perpetrator Characteristics Scale	6 items on frequency of victimization in locations, i.e., inside or outside of school. 3 items on characteristics of perpetrator, i.e., older or more powerful. Point values are assigned to responses: never=0; once=1; a few times=2; many times=3.	These items were not considered a subscale and alpha was not calculated.
Peer Victimization Impact	6 items on frequency of impact of peer victimization, i.e. feeling sick, not able to study. Point values are assigned to responses: never=0; once=1; a few times=2; many times=3. Scale scores summed to a possible range of 0 to 18 for impact of victimization.	Impact of victimization=0.603
**Child Mental Health**		
Children's Depression Inventory 2 (CDI 2) ^23^	28-item self-report questionnaire to assess the severity of current or recent (last 2 weeks) depressive symptoms. Response options are rated on a 3-point scale as: 0=no symptom; 1=mild symptom; 2=definite symptom. Scale scores range from 0 to 56.	Alpha=0.725
**Investigator-Derived Questions**		
Corporal Punishment at School	6 items on the frequency (i.e., never, once, 2–3 times, or 4 or more times) the youth was punished by a teacher (i.e., slapped, hit or beaten, made to run, kneel or stand). Scale scores range from 0 to 24.	Alpha=0.758
Parent Fighting	3 items on frequency (i.e., never, once, 2–3 times, or 4 or more times) child witnessed parent fighting, including father violence against the mother, father violence against other adults, mother violence against other family members.	These items were not considered a subscale and alpha was not calculated.
Child Attitudes Toward Child Punishment	5 items that assess child agreement (i.e., strongly agree, agree, disagree, and strongly disagree) with events that deserve child punishment, such as disobeying parents and misbehaving at school. Scale scores range from 0 to 15.	Alpha=0.653
Child Attitudes Toward Gender Norms and Women's Participation	13 items that assesses child agreement (i.e., strongly agree, agree, disagree, and strongly disagree) with gender norms, such as girls going to school, wives obeying husbands, husbands' right to punish wives, and women's participation in social events and employment. Scale scores range from 0 to 39.	Alpha=0.738
Child Physical Punishment at Home	2 items to assess parental physical punishment frequency (i.e., never, once, 2–3 times, 4 or more times) and severity to the child at home.	Due to only 2 items, coefficient alpha was not determined.
Family Life	9 items that assess food security, parent literacy, and home assets, such as electricity and water.	Due to many of the items having a dissimilar metric and dichotomous responses, coefficient alpha was not determined.
Early Marriage	3 items that assess if the child has been promised in marriage and age of marriage of older siblings.	Due to only 2 items having a similar metric, coefficient alpha was not determined.
Child School Performance	7 items that assess academic performance (i.e., below average, average, above average), number of absences from school, and reasons for absences.	Alpha=0.642, for the 4 academic performance items that had a similar metric.

### Procedures

#### Pilot Testing

Following review and approval by the Ethical Review Committee of Aga Khan University and the Ethics Committee of the Medical Research Council of South Africa, we collaborated with Right To Play organization to identify fairly demographically homogenous public schools for girls and boys, who were ages 11–12 years and in the 6th grade, who would be receptive to pilot testing the instruments. The schools also had to be in a school district geographically distant from the main study site to avoid any potential for contamination. Schools with receptive school directors and willing teachers were identified. Children in the segregated girls' and boys' schools were given parental consent forms, which when returned with parent consent enabled the researchers to obtain assent from the youth. Over 90% of the parents signed consent forms and all youth assented to the questionnaire.

The instruments were tested among 124 youth attending the 6th grade who were between ages 11 and 12. The instruments were intended to be self-administered using a paper-and-pencil version. Although the instruments were written to a 6th grade reading level, many children had difficulty reading the questionnaires. Consequently, the researchers read each question, resulting in a 2.5-hour administration period. The questionnaires were revised to reduce their length, and the interview protocol was revised so that questionnaires could be self-completed but with interviewer assistance by reading the questions aloud. This enabled the questionnaires to be completed within an hour.

#### Data Collection

Data were collected at baseline during November and December 2015 and will also be collected 12 months after baseline (midpoint) and 24 months after baseline (endline). Data collection for the 40 schools, with 1 facilitator who read each question to a group of 4 children, required a team of 40 data collectors, each of whom was bilingual in the national language of Urdu and the local district language of Sindhi.

Baseline data collection for the 40 schools was completed over a 60-day period, following receipt of parental informed consent and child assent. For the 40 schools, we sent home a total of 2,486 parental consent forms and received 1,858 affirmed parent consents for a return rate of 75%. Of the 1,858 forms signed and returned by the parents, 1,767 children assented for a rate of 95%. In general, more parents of girls consented than parents of boys (79% compared with 70%, respectively). A total of 1,752 youth questionnaires were completed and entered into an SPSS database.

#### Contact List for Retention

Integral to any longitudinal study is participant retention. To minimize attrition, a contact list was formed following a protocol for retaining abused women.^24^ In addition to the home address, children were asked for parent and relative names and phone numbers so they could be contacted if they were not attending school at the time of the follow-up interviews. Participant contact details are kept under lock and key in the research office and have not been entered into a computer. Thus, there is no electronic way of connecting participant information and questionnaire responses.

Our protocol for tracking loss to follow-up is that if children are unavailable for the 12-month or 24-month follow-up interview, we will try to learn why. First, we will ask the teacher if the unavailable children are currently absent but normally present at school. If teachers respond that the children are usually present, we will make up to 3 return visits to the school to complete missing interviews. If we are unable to find the children after 3 visits, we will note the children as absent but still in school. If children are not in school, we will need to determine if the absence is due to marriage or wedding preparations, whether the children are now out of school or have transferred to another school, or whether there is another reason, such as loss of interest or refusal to attend school due to violence. To discover the reason, we will first ask the teacher and then ask the relatives or neighbors, named on the tracking form. If all strategies fail, we will visit the children's home to ask their parents.

#### Intervention Fidelity Monitoring

The primary responsibility for monitoring the fidelity of the intervention delivery rests with the intervention organization, Right To Play. To ensure fidelity, Right To Play monitors logs that record the dates a coach goes to a school, the game(s) played with grade 6 students, and the number of children from grade 6 participating in the intervention. This information is reported quarterly. The information is compared with the planned intervention delivery schedule and deviations flagged and sent to Right To Play for correction.

The research team is also conducting spot checks on the fidelity logs to ensure accuracy. Two research staff members are visiting each intervention school monthly on a randomly chosen day and independently collecting data on the work in the school over the previous month, including the number of days coaches came and the games played. To collect this information, research staff talk to a teacher and 3 randomly chosen grade-6 pupils.

#### Health and Personal Safety Protocols

Potable water and basic sanitation are major challenges in Pakistan. The geographic area of the 40 schools is located in an arid and very hot area, where temperatures of 49°C (120°F) are common. Clean drinking water and toilets are scarce at the public schools. Additionally, the journey from the University where the researchers work to the intervention schools is 3 hours each way with meager facilities for water and sanitation in transit, requiring the researchers to transport ample potable water, food, and emergency supplies for the 6-hour road journey and 6- to 8-hour work day at the schools. The 12- to 14-hour workday requires us to maintain close vigilance on hydration and fatigue level of all personnel.

For optimum safety, we follow the University safety protocol, which requires registering each trip with the University Department of Safety that assesses the level of terrorism daily and authorizes (or denies) each trip. It is not uncommon to plan and prepare for a data collection day only to be denied travel authorization, which requires cancellation with data collectors, schools, and community partners. Close adherence to health and safety was maintained throughout baseline data collection. We experienced no threats or known risks to personal safety and all staff remained hydrated.

#### Ethics

As mentioned previously, the Ethical Review Committee of Aga Khan University and the Ethics Committee of the Medical Research Council of South Africa approved this study. We used a multi-layer consent process. Each school principal was given an information sheet and asked to give written consent to the school's participation, including the randomization process.

Following school selection and consent from the principal, the research team met with the teachers at the school and established a day to send notices home with selected students regarding the parent's consent for child participation. Students were asked to submit their parent consent forms prior to participation. Many parents were illiterate, but previous research from Aga Khan University has shown that there is usually a relative living in the home who is literate to grade 7–8 and can read an information sheet to parents and help them sign consent. In addition, when distributing the informed consent forms to children, the forms were reviewed with the children to ensure the child could assist with reading. The researchers acknowledge this as a limitation and not ideal. However, our resources prohibited individual home visits to read the consent forms to parents or to use audio-recorded devices to be taken to the home due to safety concerns. After sufficient parental consent forms were received for a school, the research team placed the students with parental consent in a room and provided information to enable written consent to be given by the grade-6 students. Consenting students were asked to complete the tracking form and then the questionnaire. All participants are given study codes and only these are used on the questionnaires.

We recognize that the area of research on violence can generate an emotional response from research participants, possibly as a result of recalling their experiences of violence. Field staff was trained to provide immediate emotional support, and we provided back-up counseling from a psychologist if needed. The research protocol stated that should emotional responses (i.e., crying, becoming distraught) occur in the middle of a questionnaire, the questionnaire completion should be postponed. No compensation is given in the form of cash or gifts. However, refreshments (e.g., fruit and juice) are served each time participants complete the questionnaire.

In this study, we are particularly concerned about 2 serious adverse events: death and hospitalization for injury due to interpersonal violence. Our intervention is aimed at prevention of violence and so it is essential that we fully ascertain severe injury due to violence. We have asked schools to notify us if any of these occur to students at the study schools, and we will conduct a verbal autopsy on every death by having a trained nursing professional study team member visit the child's home. We will seek in the verbal autopsy any evidence that the death could have been linked to the intervention (in the intervention schools) or research. These events will be reported to the Ethical Review Committees of Aga Khan University and the Medical Research Council of South Africa as adverse events or serious adverse events indicating whether they are related or unrelated to study participation. Finally, and very importantly, our intervention partner Right To Play practices a child safeguarding policy that demonstrates its commitment to the welfare of children including treating all children equally, encouraging positive discipline strategies, and ensuring confidentiality (see supplementary material).

#### Baseline Data Cleaning and Validation

A validation check was conducted between the first baseline data entry and the second baseline data entry to observe possible discrepancies and to confirm them with actual item responses from the questionnaires. Once validation of the second dataset was complete, data preparation and assumptions testing was conducted.

#### Baseline Data Analysis

The analysis for the baseline study consisted of frequencies, percentages, and means and standard deviations of participant demographics and subscales stratified by gender in the intervention and control arms. Peer victimization and perpetration were also categorized using thresholds suggested by the U.S. Centers for Disease Control and Prevention (CDC) guidelines.^1^ These guidelines define a participant score on the Peer Victimization Scale or Peer Perpetration Scale of 0 to 1 as low violence and 2 or greater as high violence.

Because this study used a randomized cluster design, sample design effects were taken into consideration when analyzing the data and the statistical analysis was treated accounting for school as a cluster. Standard errors (SE) for the means/proportions accounting for the sample design are presented in the subsequent tables. The analysis of the subscales provided comparisons of intervention and control groups by gender testing for significant differences between intervention and control to establish whether randomization was successful. A multivariate test of each of the subscales was conducted to observe the independent effects of gender, treatment, and their interaction using random effects linear regression to account for the cluster effect of school. This trial data will be analyzed to assess whether the intervention was successful in subsequent studies. Pairwise comparisons of marginal linear predictions were conducted to evaluate multivariate group comparisons of intervention and control arms within gender groups.

## BASELINE RESULTS

Background characteristics of the full sample are outlined in Table 3, specific to intervention arm and gender. On average, participants in each study group were between 12 to 13 years old, and the majority were 12 years old. We might have expected younger students to enroll in our study since eligibility criteria focused on grade-6 students, but most of the participants may have been older due to failing exams, which often occurs due to missing many school days. The mean number of people who lived in a household ranged from about 9 to 10 people for all groups. The mean number of brothers ranged between 2 to 3 for all groups as did the mean number of sisters.

**TABLE 3.** Background Characteristics of Study Participants by Gender and Study Arm, Hyderabad, Pakistan, 2016

	Boys		Girls	
	Intervention	Control	Intervention	Control
Age				
N	446	375	480	447
Mean	12.53	12.49	12.16	12.39
SE	0.06	0.11	0.11	0.15
No. of people living in the home				
N	446	376	481	447
Mean	9.96	9.21	9.65	10.30
SE	0.20	0.47	0.27	0.41
No. of brothers				
N	443	376	483	447
Mean	2.77	2.61	2.21	2.21
SE	0.13	0.12	0.08	0.10
No. of sisters				
N	442	374	483	447
Mean	2.25	2.13	2.57	2.70
SE	0.07	0.10	0.13	0.13

Abbreviation: SE, standard error.

Means and standard deviations of the primary outcome measures for the full sample are shown in Table 4, specific to intervention arm and gender. Boys showed a much higher prevalence for both peer victimization and peer perpetration than girls. For example, the average score for peer victimization among boys in the intervention arm was 12.32 (SE=0.58) compared with 7.89 (SE=0.47) among girls in the intervention arm. Boys had higher mean scores for child depression as well (11.07 [SE=0.24] for boys in the intervention arm vs. 9.52 [SE=0.43] for girls in the intervention arm). Additionally, boys reported higher negative mood and self-esteem scores compared with girls as well as more interpersonal and emotional problems. There was little difference between the intervention and control arms within gender groups. Independent sample *t* tests were conducted to observe the difference of means between intervention and control groups within gender. The results revealed that in most cases there was group equivalence across intervention and control groups within gender, all values of *P*<.05.

**TABLE 4.** Primary Outcome Measures Related to Peer Violence by Gender and Intervention and Control Arms, Hyderabad, Pakistan, 2016

	Boys		Girls	
	Intervention	Control	Intervention	Control
Peer victimization scale sum				
N	422	370	462	434
Mean	12.32	12.75	7.89	6.32
SE	0.58	0.89	0.47	0.60
Peer perpetration scale sum				
N	428	369	468	442
Mean	7.42	7.27	3.48	2.85
SE	0.48	0.55	0.40	0.28
Peer victimization impact scale sum				
N	435	372	482	438
Mean	3.91	3.48	3.07	2.46
SE	0.20	0.24	0.28	0.23
CDI 2 scale				
N	445	373	481	443
Mean	11.07	10.97	9.52	8.79
SE	0.24	0.44	0.43	0.32
CDI 2 Total T-score				
N	445	372	478	443
Mean	56.87	56.60	55.40	53.75
SE	0.40	0.73	0.84	0.63

Abbreviations: CDI, Children's Depression Inventory; SE, standard error.

Boys reported higher prevalence of peer victimization and perpetration than girls, as well as poorer scores on a number of mental health measures.

The Figure illustrates the percentage of participants in the intervention and control arms by gender that reported low- and high-violence perpetration and victimization at baseline before the intervention began. Based on the youths' reports and using the CDC cutoffs of 2 or more acts of violence perpetration or victimization as high-violence, the large majority of boys across study groups fell into the high-violence categories for both peer victimization and perpetration. Most girls also fell into the high-violence categories. Among the total sample of 1,752 youth (intervention and control groups combined) asked about victimization or perpetration of violence within the preceding 4 weeks, 94% of the boys and 85% of the girls reported 1 or more episodes of victimization, with almost identical reporting percentages between intervention and control groups. Regarding perpetration of violence, 85% of the boys and 66% of the girls endorsed 1 or more behaviors of perpetration, again with almost identical reporting between intervention and control groups. Additional analyses revealed that within the boys group, 15.7% of the participants in the intervention arm and 13.6% in the control arm reported no perpetration. Within the girls group, 32.3% in the intervention arm and 35.7% in the control arm reported no perpetration. Conversely, within the boys group, only 5% in the intervention arm and 6.9% in the control arm experienced no victimization. Within the girls group, 14.3% in the intervention arm and 15.9% in the control arm experienced no victimization.

94% of boys and 85% of girls reported 1 or more episodes of peer victimization, and 85% of boys and 66% of girls reported perpetrating at least 1 of the same behaviors.

**FIGURE:**
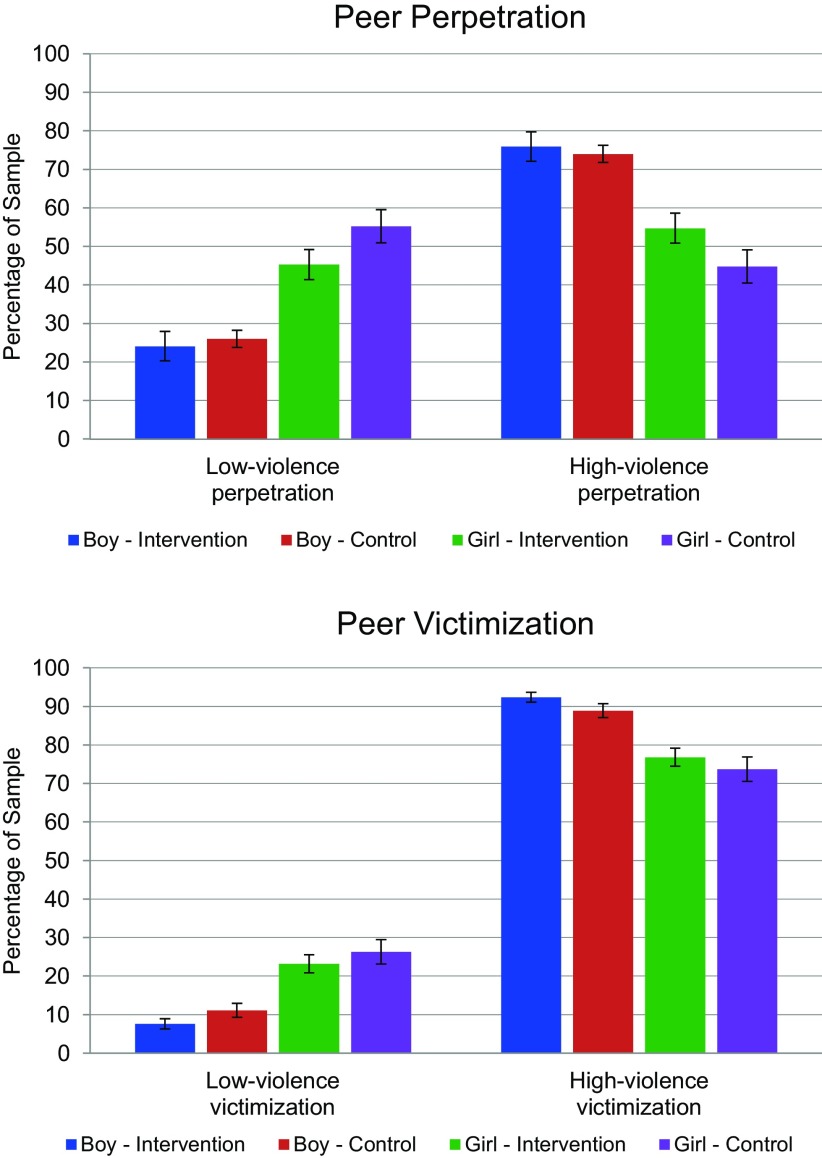
Percentage of Participants Exposed to Low-Violence and High-Violence Peer Perpetration and Victimization Using CDC Cutoffs^a^ by Study Arm and Gender, Hyderabad, Pakistan, 2016

Abbreviation: CDC, U.S. Centers for Disease Control and Prevention.

Note: Error bars represent cluster design effect standard errors.

^a^ Participant score of 0–1 is defined as low violence and ≥2 as high violence.

Frequencies and percentages for specific types of peer victimization by gender are presented in Table 5. It is clear that a gender difference exists for each type of victimization. Across all 16 items of the peer victimization instrument, boys reported experiencing greater frequency of types of victimization than girls while girls reported a higher percentage of the never frequency category across these items relative to boys. Pearson's chi-square tests of association confirmed these gender differences for all items (*P*<.05).

**TABLE 5.** Frequencies and Percentages of Types of Peer Victimization Items by Gender, Hyderabad, Pakistan, 2016

Types of Peer Victimization	Boys, No. (%)	Girls, No. (%)
Called me bad names		
Never	387 (47.1)	596 (64.1)
Once	156 (19.0)	157 (16.9)
A few times (2 or 3)	128 (15.6)	86 (9.2)
Many times (4 or more)	149 (18.1)	91 (9.8)
Tried to get me into trouble with my friends		
Never	458 (55.7)	707 (76.0)
Once	176 (21.4)	128 (13.8)
A few times (2 or 3)	128 (15.6)	53 (5.7)
Many times (4 or more)	57 (6.9)	38 (4.1)
Took something of mine without permission		
Never	350 (42.6)	486 (52.3)
Once	208 (25.3)	196 (21.1)
A few times (2 or 3)	133 (16.2)	132 (14.2)
Many times (4 or more)	126 (15.3)	112 (12.0)
Made fun of me because of my appearance		
Never	544 (66.2)	762 (81.9)
Once	132 (16.1)	97 (10.4)
A few times (2 or 3)	88 (10.7)	43 (4.6)
Many times (4 or more)	54 (6.6)	26 (2.8)
Made fun of me for some reason apart from my appearance		
Never	490 (59.6)	713 (76.7)
Once	181 (22.0)	124 (13.3)
A few times (2 or 3)	95 (11.6)	61 (6.6)
Many times (4 or more)	56 (6.8)	24 (2.6)
Tripped me to make me fall		
Never	385 (46.8)	689 (74.1)
Once	223 (27.1)	167 (18.0)
A few times (2 or 3)	134 (16.3)	46 (4.9)
Many times (4 or more)	77 (9.4)	28 (3.0)
Pushed me to hurt me		
Never	399 (48.5)	634 (68.2)
Once	205 (24.9)	173 (18.6)
A few times (2 or 3)	139 (16.9)	80 (8.6)
Many times (4 or more)	78 (9.5)	40 (4.3)
Hurt me physically		
Never	497 (60.5)	720 (77.4)
Once	189 (23.0)	145 (15.6)
A few times (2 or 3)	85 (10.3)	43 (4.6)
Many times (4 or more)	50 (6.1)	19 (2.0)
Beat me so badly that I was injured		
Never	669 (81.4)	850 (91.4)
Once	80 (9.7)	49 (5.3)
A few times (2 or 3)	41 (5.0)	15 (1.6)
Many times (4 or more)	30 (3.6)	12 (1.3)
Deliberately broke something that belongs to me		
Never	462 (56.2)	653 (70.2)
Once	214 (26.0)	183 (19.7)
A few times (2 or 3)	88 (10.7)	65 (7.0)
Many times (4 or more)	56 (6.8)	26 (2.8)
Tried to make other children turn against me		
Never	376 (45.7)	557 (59.9)
Once	218 (26.5)	195 (21.0)
A few times (2 or 3)	126 (15.3)	93 (10.0)
Many times (4 or more)	99 (12.0)	84 (9.0)
Stole something from me		
Never	398 (48.4)	608 (65.4)
Once	221 (26.9)	168 (18.1)
A few times (2 or 3)	115 (14.0)	84 (9.0)
Many times (4 or more)	85 (10.3)	63 (6.8)
Refused to talk to me		
Never	478 (58.2)	597 (64.2)
Once	178 (21.7)	199 (21.4)
A few times (2 or 3)	97 (11.8)	83 (8.9)
Many times (4 or more)	66 (8.0)	48 (5.2)
Made other people not talk to me		
Never	485 (59.0)	655 (70.4)
Once	169 (20.6)	141 (15.2)
A few times (2 or 3)	108 (13.1)	83 (8.9)
Many times (4 or more)	58 (7.1)	50 (5.4)
Deliberately damaged something of mine		
Never	580 (70.6)	766 (82.4)
Once	142 (17.3)	94 (10.1)
A few times (2 or 3)	60 (7.3)	43 (4.6)
Many times (4 or more)	38 (4.6)	25 (2.7)
Swore at me		
Never	241 (29.3)	642 (69.0)
Once	168 (20.4)	143 (15.4)
A few times (2 or 3)	138 (16.8)	72 (7.7)
Many times (4 or more)	274 (33.3)	73 (7.8)

Frequencies and percentages for types of peer perpetration by gender are presented in Table 6. The results mirror the gender differences of peer victimization, with boys reporting greater frequency and type of perpetration relative to girls and girls reporting a higher percentage of the never category relative to boys. Pearson's chi-square tests of association confirmed these gender differences for all items(*P*<.05).

**TABLE 6.** Frequencies and Percentages of Types of Peer Perpetration by Gender, Hyderabad, Pakistan, 2016

Types of Peer Perpetration	Boys, No. (%)	Girls, No. (%)
Called another child bad names		
Never	373 (45.4)	614 (66.0)
Once	243 (29.6)	214 (23.0)
A few times (2 or 3)	118 (14.4)	70 (7.5)
Many times (4 or more)	87 (10.6)	30 (3.2)
Tried to get another child into trouble with friends		
Never	629 (76.5)	837 (90.0)
Once	119 (14.5)	68 (7.3)
A few times (2 or 3)	44 (5.4)	17 (1.8)
Many times (4 or more)	29 (3.5)	7 (0.8)
Upset or annoyed another child by taking something of theirs without permission		
Never	567 (69.0)	749 (80.5)
Once	152 (18.5)	138 (14.8)
A few times (2 or 3)	67 (8.2)	31 (3.3)
Many times (4 or more)	35 (4.3)	11 (1.2)
Made fun of another child because of their appearance		
Never	518 (63.0)	760 (81.7)
Once	192 (23.4)	126 (13.5)
A few times (2 or 3)	86 (10.5)	30 (3.2)
Many times (4 or more)	26 (3.2)	13 (1.4)
Made fun of another child for some reason apart from their appearance		
Never	506 (61.6)	733 (78.8)
Once	179 (21.8)	145 (15.6)
A few times (2 or 3)	107 (13.0)	37 (4.0)
Many times (4 or more)	29 (3.5)	14 (1.5)
Tripped another child to make him or her fall		
Never	551 (67.0)	809 (87.0)
Once	185 (22.5)	90 (9.7)
A few times (2 or 3)	61 (7.4)	20 (2.2)
Many times (4 or more)	25 (3.0)	10 (1.1)
Pushed another child to hurt him or her		
Never	581 (70.7)	798 (85.8)
Once	151 (18.4)	104 (11.2)
A few times (2 or 3)	56 (6.8)	20 (2.2)
Many times (4 or more)	31 (3.8)	7 (0.8)
Hurt another child physically		
Never	628 (76.4)	858 (92.3)
Once	122 (14.8)	55 (5.9)
A few times (2 or 3)	50 (6.1)	7 (0.8)
Many times (4 or more)	19 (2.3)	9 (1.0)
Beat another child so badly that they were injured		
Never	693 (84.3)	894 (96.1)
Once	83 (10.1)	28 (3.0)
A few times (2 or 3)	24 (2.9)	3 (0.3)
Many times (4 or more)	20 (2.4)	2 (0.2)
Deliberately broken something that belong to another child		
Never	606 (73.7)	821 (88.3)
Once	165 (20.1	85 (9.1)
A few times (2 or 3)	28 (3.4)	17 (1.8)
Many times (4 or more)	22 (2.7)	5 (0.5)
Tried to make other children turn against another child		
Never	575 (70.0)	774 (83.2)
Once	167 (20.3)	126 (13.5)
A few times (2 or 3)	55 (6.7)	22 (2.4)
Many times (4 or more)	21 (2.6)	7 (0.8)
Stolen something from another child		
Never	697 (84.8)	862 (92.7)
Once	86 (10.5)	49 (5.3)
A few times (2 or 3)	24 (2.9)	9 (1.0)
Many times (4 or more)	9 (1.1)	8 (0.9)
Refused to talk to another child		
Never	500 (60.8)	627 (67.4)
Once	207 (25.2)	243 (26.1)
A few times (2 or 3)	84 (10.2)	41 (4.4)
Many times (4 or more)	30 (3.6)	19 (2.0)
Made other children not talk to another child		
Never	572 (69.6)	777 (83.5)
Once	158 (19.2)	97 (10.4)
A few times (2 or 3)	63 (7.7)	39 (4.2)
Many times (4 or more)	27 (3.3)	15 (1.6)
Deliberately damaged something of another child's		
Never	665 (80.9)	861 (92.6)
Once	100 (12.2)	48 (5.2)
A few times (2 or 3)	33 (4.0)	15 (1.6)
Many times (4 or more)	21 (2.6)	5 (0.5)
Swear at another child		
Never	427 (51.9)	795 (85.5)
Once	198 (24.1)	86 (9.2)
A few times (2 or 3)	114 (13.9)	30 (3.2)
Many times (4 or more)	82 (10.0)	18 (1.9)

The results of Table 7 reveal that boys had significantly higher scores on each of the 5 measures relative to girls. There was a significant difference between intervention and control groups for peer victimization (β, 1.60; *P*=.04) but not for any of the other 4 measures. There was no significant interaction of gender and intervention in this regression (*P*>.05). When comparing treatment and control groups, there was only one significant comparison: peer victimization between treatment and control groups for girls (β, −1.60; *P*=.04). Mean difference testing revealed that group equivalence existed between intervention and control and that the randomized cluster sample design was successful.

**TABLE 7.** Random Effects Linear Regression Model of Gender, Study Group, and their Interaction Predicting Key Peer Violence Scales With Pairwise Comparisons, Hyderabad, Pakistan, 2016

Predictors	Peer Victimization		Peer Perpetration		Victimization Impact		Child Depression		Child Depression T-Score	
	β	*P*	β	*P*	β	*P*	β	*P*	β	*P*
Gender, Boy (ref: female)	6.3	<.001	3.78	<.001	0.95	0.008	2.13	<.001	2.85	0.002
Group, Intervention (ref: control)	1.6	0.04	0.61	0.19	0.62	0.09	0.81	0.12	1.69	0.08
Interaction:	−1.79	0.17	−0.15	0.86	−0.08	0.87	−0.6	0.4	−1.3	0.31
Gender & Group (ref: female & control)										

Group Comparisons^a^	Contrast	*P*	Contrast	*P*	Contrast	*P*	Contrast	*P*	Contrast	*P*
Boys: Control vs. Intervention	0.19	0.86	−0.47	0.51	−0.54	0.09	−0.21	0.68	−0.39	0.64
Girls: Control vs. Intervention	−1.6	0.04	−0.61	0.19	−0.62	0.09	−0.81	0.12	−1.69	0.08

Note: All coefficients reported are unstandardized betas.

a Pairwise comparisons of marginal linear predictions

## DISCUSSION

Although we identified no studies measuring both youth victimization and perpetration among school-age youth, ages 12 to 14, with which we could compare our results, our prevalence of 89% of youth reporting peer victimization far exceeds global estimates of 50%.^1^ Equally high is our finding of 75% of the youth perpetrating peer violence within the preceding 4 weeks. Although measurement time for victimization varies, other studies of school-age youth consistently report appreciably lower prevalence of victimization than we found, and the victimization is frequently defined as bullying.

For example, in the Global School-Based Health Survey for Pakistan completed in 2009 by the Ministry of Health in collaboration with the World Health Organization and the CDC, among students in grades 8–10 (slightly older than the youth in our study), overall prevalence of bullying victimization in the past 30 days was 41.3%.^25^ The prevalence was 45.1% among male students and 35.5% among females. Loneliness and sleep disturbance were significantly more common among youth reporting bullying.^25^ The same Global School-Based Health Survey question on bullying was administered in Thailand to youth in grades 7–9, revealing an overall prevalence of bullying of 27.8% (32.9% among males and 23.2% among females). Youth who reported bullying were more likely to also report psychosocial problems.^26^

When 2,264 adolescents in Malawi were surveyed about bullying in a school health survey, almost equal percentages of boys and girls (44% and 45%, respectively) reported being bullied.^27^ However, among a sample of 1,559 school-age youth in grades 7–10 in Zambia, more girls (65%) than boys (60%) reported being bullied in the past 30 days.^28^

Irrespective of prevalence, all these studies found appreciably higher psychological problems, such as anxiety, worry, and eating and sleeping disorders, among youth reporting victimization. In our study, boys reported appreciably more depression compared with girls as well as higher negative mood and self-esteem scores and more interpersonal and emotional problems. These gender differences and associations with health and functioning will be explored in future papers.

**Figure:**
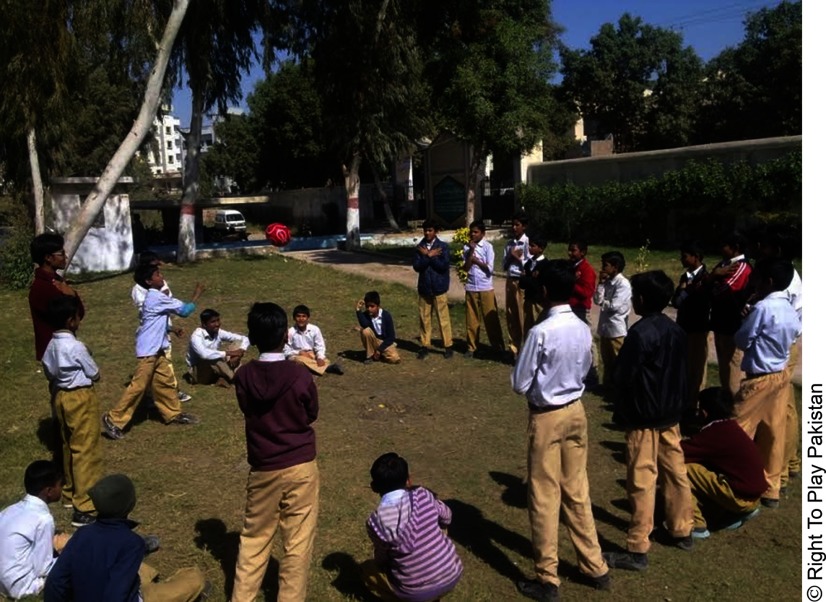
The Right To Play program in Pakistan focuses on 4 areas of child and youth development including physical, cognitive, social, and emotional components. At baseline, boys reported more depression and interpersonal and emotional problems than girls.

### Limitations

The study is designed to evaluate Right To Play's intervention, but we can only test the intervention in one of the many countries in which it is delivered. We cannot generalize our results to the whole of Pakistan, all school grades, or all Right To Play programs. However, the Pakistan program is very large and has been underway for over a decade, and so a rigorous evaluation is timely. This is an effectiveness trial and so we are monitoring fidelity to the intervention and report this information to the implementers, but in other respects we are not able to influence the fidelity of the intervention.

Pakistan may be a particularly challenging setting for evaluating Right To Play's intervention. For example, there is a serious problem in Pakistan of children not being able to attend school regularly, which impacts all school-delivered interventions. We ask about attendance in the questionnaire and important reasons for lack of attendance are lack of money for transport and a need for the children to engage in income-generating activities. There are also some difficulties with play-based activities when it is very hot, as for some months of the year it is over 40°C. Lack of food and drinking water for children also influences participation and attendance, and some children, both boys and girls, leave school early due to lack of school toilets. Days are also often lost from school due to severe weather during the monsoon, and there is often a delay in return to school after holidays. There may also be teachers' strikes. These factors will have an impact on the dose of Right To Play intervention that is delivered, and therefore considerable caution will be needed in generalizing from the study findings.

Children with disabilities are admitted to special public schools where their physical and/or emotional challenges can be accommodated and therefore are not part of this study. However, to learn if there are children with disabilities in public schools, we will ask in future interviews a series of questions related to disabilities.

Our research methodology has limitations that it may under- or overrepresent victimization, perpetration, and the functioning outcomes of the child participants. The questions may miss some episodes of victimization or perpetration and incorrectly classify others, particularly with respect to the 4-week reporting period. Children may not accurately recall the timing and type of victimization or perpetration they experienced (i.e., whether or not the exposure occurred within the last 4 weeks). The researchers acknowledge recall bias is operant in all questions.

The questionnaire focuses on a small number of measures that we believe can be more accurately recalled and reported by children and more reliably measured. This results in our failing to collect information on other impacts of the intervention. Further, the choice of a 24-month endline prevents us from studying sustainability or attrition of effect post-endline. Finally, our participants were limited to Sindhi and Urdu speakers, although these are the languages of teaching in the participating schools.

Despite these limitations, the researchers feel this study provides a framework for understanding impact of the Right To Play intervention and the most detailed and comprehensive data available on the frequency and severity of peer perpetration and victimization of grade-6 male and female children in urban public schools in Pakistan as well as associated gender attitudes and family life.

## CONCLUSIONS

Some 89% of 6th-grade youth attending public schools in an urban area of Pakistan reported peer victimization within the last 4 weeks and 75% reported they perpetrated violence within the same time period. Evidence confirms violence against children transfers to poor health and increased mortality in adulthood as well as use of violence against women.^3^^,^^4^ If girls are sexually abused in childhood, the risk for intimate partner violence doubles,^29^ and women abused during pregnancy are at high risk for pregnancy complications, fetal demise, and low birthweight offspring.^30^ The intergenerational impact of abuse escalates from violence against children to traumatized mothers to dysfunctional offspring.^31^^,^^32^

Violence against children transfers to poor health and increased mortality in adulthood as well as use of violence against women.

More than 1 billion youth, 50% of the world's population, are victimized each year and more than one-third of all adult women experience violence.^33^ Building the global evidence base for prevention of violence against children and women is critical if we are ever to be able to eradicate these problems and allow children and women to reach their full social and economic potential and optimal emotional well-being. In the medium-term, contributing to this evidence base enables optimum progress toward the 2030 Sustainable Development Goals.^6^ This evaluation, scheduled to be completed in 2018, is poised to make an important contribution as Right To Play already has a large global footprint and extensive exposure among the more than 190 million people who live in Pakistan. Further, the intervention has the potential for enabling the next generation of young Pakistanis to live more empowered and peaceable lives, which is an incredibly important goal in a country that has been wracked by decades of political, religious, and criminal violence.

## Notes

**Peer Reviewed**
